# Involvement of Visual Mismatch Negativity in Access Processing to Visual Awareness

**DOI:** 10.3389/fnhum.2021.757411

**Published:** 2021-11-05

**Authors:** Yuki Kurita, Tomokazu Urakawa, Osamu Araki

**Affiliations:** Department of Applied Physics, Faculty of Science, Tokyo University of Science, Tokyo, Japan

**Keywords:** visual mismatch negativity, event-related potential, visual awareness, unconscious processing, binocular rivalry, fast visual processing

## Abstract

Psychophysiological studies with electroencephalography, focusing on the dynamical aspect of neural correlate of consciousness, reported that visual awareness negativity and P3 enhancement are observed at a latency, 200–300 ms after the visual stimulus onset, when the visual stimulus is consciously perceived. However, access processing to visual awareness (APVA) immediately before conscious perception still remains at the earlier stage of visual sensory processing, though there is little known regarding this subject. The present study hypothesized that visual mismatch negativity (vMMN), which reflects automatic change detection at a latency of 130–250 ms, might be involved in the APVA. In a previous study, vMMN was reported to be evoked by the deviant stimulus that is not consciously perceived in binocular rivalry. To clarify whether the visual change detection affects APVA, we conducted a modified experiment of oddball paradigm on binocular rivalry. The results showed a significant correlation between enhancement of vMMN amplitude and facilitation of perceptual alternation when the unconscious deviant was presented. This implies that vMMN is relevant to the APVA, which is a novel role of vMMN. In early visual processing, the attentional mechanism associated with vMMN is suggested to play an important role in unconscious neural processing at an earlier stage of visual awareness.

## Introduction

Previous neuropsychological studies on visual awareness have been trying to elucidate neural processes that are correlated with the subjective conscious experience, analyzing the differences in neural activities between when the visual stimulus is consciously perceived and when it is not consciously perceived (Koivisto and Revonsuo, 2010; Dehaene and Changeux, 2011; Aru et al., 2012; Silverstein et al., 2015; Koch et al., 2016). Studies of the neural correlate of consciousness (NCC) using functional magnetic resonance imaging (fMRI), indicated that the posterior cortical areas are related to the neural mechanism of visual awareness (e.g., Lumer et al., 1998; Polonsky et al., 2000; Lee et al., 2005; Frässle et al., 2014). On the other hand, studies using electroencephalography (EEG) focusing on the temporal aspect of NCC indicated that visual awareness negativity (VAN) and P3 enhancement were observed at a latency of about 200 and 300 ms after visual stimulus onset, respectively, when the visual stimulus was consciously perceived (for review: Koivisto and Revonsuo, 2010; Förster et al., 2020). In particular, VAN is one of the earliest brain activities related to visual awareness and is often used as an index of visual awareness (Koivisto and Revonsuo, 2003, 2010; Koivisto et al., 2009; Pitts et al., 2014; Eklund and Wiens, 2018). On the other hand, from the viewpoint of whether the processing is conscious or unconscious, some studies reported that VAN does not reflect unconscious neural processing, rather it presents the stage of graded visual consciousness (Koivisto and Grassini, 2016; Koivisto et al., 2017).

The present study hypothesized a type of unconscious neural processing that determines whether the unconscious stimulus is consciously perceived or not [hereafter, we call the neural processing as access processing to visual awareness (APVA)] at the earlier stage of visual sensory processing than the emergence of VAN. According to previous studies (Prinz, 2011; Bor and Seth, 2012; Cohen et al., 2012), attentional mechanisms would impinge on visual processing earlier than the emergence of VAN; the attentional mechanism would contribute to APVA. These attentional mechanisms were considered to be restricted to those accompanied by visual processing earlier than VAN. A previous study using an invisible Gabor stimulus indicated that attention is directed to unconscious visual stimuli and that attention makes it easier for these to be perceived consciously (Jiang et al., 2006). An abruptly-presented visual stimulus, inducing attentional capture at a certain location of the visual field, was also reported to facilitate conscious perception of a subsequent in near-threshold target image at the location (Chica et al., 2010; Chica et al., 2011). Therefore, the attentional mechanism appears to facilitate visual processing for the unconscious visual stimulus, so that the stimulus information crosses the boundary between consciousness and unconsciousness.

The present EEG study focused on visual mismatch negativity (vMMN) as one of the neural mechanisms involved in APVA. vMMN is a relative enhancement in visual evoked potential (VEP) to an infrequently presented visual stimulus (deviant) over a repetitively presented stimulus (standard). vMMN appeared over posterior electrodes at a latency of about 130–250 ms (Czigler et al., 2002; Astikainen et al., 2008; Kimura et al., 2009). This negative-going component appeared even when participants did not pay attention to the deviant, and the response enhancement was interpreted to reflect automatic visual change detection. A previous study reported that vMMN appeared when the deviant was unconsciously presented using binocular rivalry (Jack et al., 2017). In other words, visual change detection occurs even unconsciously, and it appears that vMMN, which is an earlier VEP component than VAN, originates from the unconscious neural mechanism. In addition, visual change detection is involved in the involuntary attentional orienting response to the deviant (Astikainen et al., 2008; Urakawa et al., 2010). The attentional orienting response induced by the visual mismatch process to the unconscious deviant would be relevant to the conscious perception of the deviant stimulus. However, the relationship between vMMN and behavior, in addition to conscious perception, has not yet been well-established because vMMN is merely assumed to reflect automatic visual change detection (Stefanics et al., 2014). We hypothesized that vMMN is related to the orienting of attention to the unconscious deviant, and that it is involved in APVA.

Jack et al. (2017) reported that vMMN is evoked by the unconscious deviant, but they did not clarify the effect of vMMN on conscious perception. In their experimental paradigm, the deviant stimulus was a rapid decrease in the luminance of the stimulus in binocular rivalry. Since decreasing the intensity of the luminance reduces the perceptual alternation rate (Levelt, 1965), it is impossible to fairly evaluate whether vMMN affects visual perception or not. For this reason, in the present study, we used the orientation change of the grating stimulus as the deviant that did not reduce the stimulus luminance in order to evaluate perceptual alternation on binocular rivalry.

To clarify whether visual change detection affects APVA, we conducted the modified oddball paradigm on binocular rivalry, based on previous studies (Jack et al., 2017; Urakawa et al., 2017a). Our previous study (Urakawa et al., 2017a) used the Necker cube and surrounding bars whose orientations change in the deviant condition. The major difference from our stimulation paradigm was that the deviant stimulus was presented unconsciously. Under this stimulation paradigm, we recorded vMMN and perceptual alternation in binocular rivalry. The proportion of perceptual alternation from before to after the presentations of the standard was subtracted from that from before to after the presentations of the deviant, so as to evaluate the facilitation of APVA by the deviant. In order to clarify the relationship between vMMN and APVA, we focused on inter-individual variability and then examined whether vMMN enhancement is correlated with an increase in the proportion of perceptual alternation across participants, as in our previous studies (Urakawa et al., 2017a, 2018). Such an evaluation of the relationship between behavioral and neural data with a focus on inter-individual difference is a powerful analytical approach for induction of the neural mechanisms underlying behavioral data (Vogel and Awh, 2008).

## Materials and Methods

### Participants

Nineteen healthy volunteers (19 males, age 21–36, mean ± SD, 23.2 ± 0.76 years) participated in this study. All of them were right-handed and had normal or corrected-to-normal visual acuity. Informed consent was received from all participants, and this study was approved by the ethics committee of the Tokyo University of Science.

### Stimulus and Procedure

**FIGURE 1:**
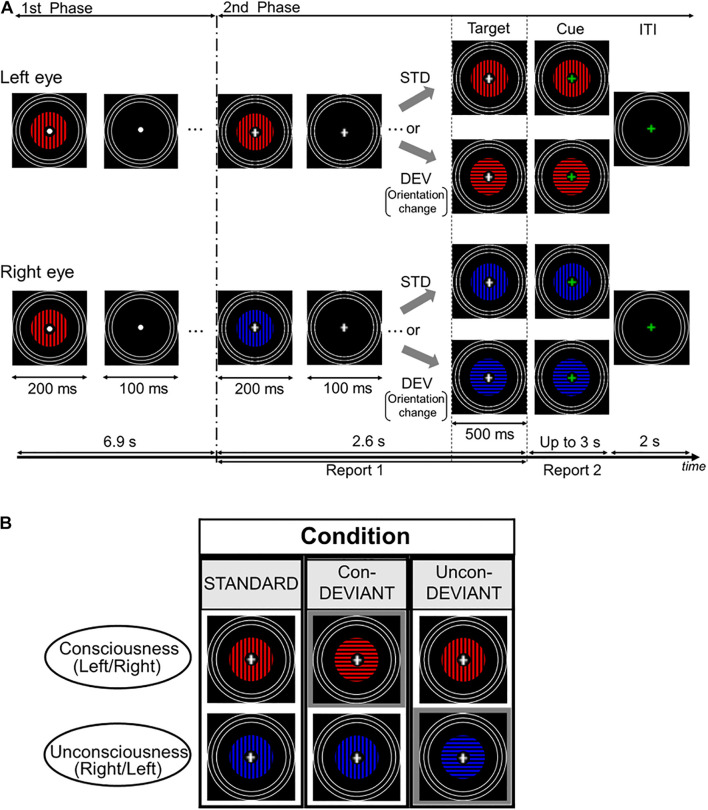
Time course of stimulus presentation in one trial and experimental conditions. **(A)** Each trial consisted of two stimulation phases. In the first phase, an identical grating stimulus with a color (blue or red) was simultaneously and intermittently presented for both the left eye and the right eye. Participants were asked to passively look at the fixation point. In the second phase, a color of the grating stimulus changed for either of two eyes to induce the binocular rivalry (a color change from blue to red or vice versa), and the grating images were presented intermittently as in the first phase. During this phase, the fixation cross was continuously presented at the central area of the gratings, and participants were required to continuously report the perceived grating. Following the second phase, the target stimulus immediately appeared for 500 ms and its fixation cross then changed in color to a green cross (the cue). Participants were asked to report the current perceived grating stimulus after the onset of the cue. **(B)** Each pear of two grating stimuli arranged vertically indicates an example of target stimulus. Target stimulus had three variants by changing or not changing an orientation of the grating stimulus under the binocular rivalry. In the Uncon-DEV condition, an orientation of the grating stimulus which did not reach a conscious percept was exclusively changed by 90°. Meanwhile, in the Con-DEV condition, orientation of the grating stimulus which reached a conscious percept was exclusively changed by 90°. In the STD condition, there was no change of the grating stimulus for both eyes.

Images were presented on a liquid crystal display (BenQ XL2540) using the MATLAB Psychophysics Toolbox (Brainard, 1997; Pelli, 1997). Participants were presented with two computer-generated images through a mirror stereoscope. The image included gratings that were annulus-shaped with a spatial frequency of 1.3 cycles/degree (Figure 1A). The outer radius of the gratings was 4.3°, and the inner radius was 0.57°. A white fixation point was presented at the center of the grating image. The blue or red grating was presented on a black background with a mean luminance of 0.05 cd/m^2^. The mean luminance of the grating’s red portion was 3.56 cd/m^2^, while that of the blue portion was 2.16 cd/m^2^. Each grating was surrounded by three white rings, which served to lock vergence. Every white ring had a line width of 0.19°. The outer radius of the largest ring was 8.64°, and the outer edges of each of the other two smaller rings were inwardly depicted by 0.64° from the outer edge of a neighboring larger ring. The white rings for both eyes were continuously presented throughout the period of stimulation.

Figure 1A shows the procedure of the stimulation for one trial. Based on a stimulation scheme used in our previous studies (Urakawa et al., 2017a,b, 2018), each trial had two consecutive phases. In the first phase, an identical grating image was simultaneously presented for both left and right eyes. The grating images were intermittently presented 23 times, with a duration and the inter-stimulus interval (ISI) of 200 and 100 ms each, respectively. The grating was either blue or red, and its orientation was either horizontal or vertical. Between these images, an image without grating was presented for 100 ms in both eyes. In the presentation of the grating image, the colors were counterbalanced across trials for each participant. An orientation of the grating in the first phase was kept constant for each participant, and the orientation was counterbalanced among participants. In the first phase, participants were asked to passively look at the fixation point. Immediately following the first phase, in the second phase, the grating image used in the first phase was manipulated to induce binocular rivalry by changing its color from blue to red or vice versa for either of the two eyes, at the beginning of the second phase. The occurrence of the color change was counterbalanced between the two eyes. Similar to the first phase, the gratings in the second phase were simultaneously and intermittently presented, without changing the grating image for each eye. The duration and the ISI used in the second phase were the same as those in the first phase. The duration and ISI were expected to mitigate binocular fusion (Wolfe, 1983). According to previous psychophysiological studies (e.g., Astikainen et al., 2008; Kimura et al., 2009; Urakawa et al., 2017a), vMMN was also expected to appear in the duration and the ISI. In the intermittent stimulation for the second phase, the gratings were presented seven times. During the second phase, the white fixation cross appeared at the center of the grating image instead of the white fixation point. When the white cross appeared (i.e., when the second phase started), participants were required to fixate on the cross and to press a key on the keyboard in front of them to start reporting a perceived grating image. In this behavioral task, participants were asked to continuously press the left arrow key during a period in which the blue grating was perceived, or to continuously press the right arrow key during the period in which the red grating was perceived. Meanwhile, during the period in which the blue and the red gratings merged in perception, participants were instructed not to press any key. This behavioral task continued throughout the second phase. In every trial, participants were asked to keep their initial percepts during the second phase as much as possible. The second phase ended with the blank image lasting for 100 ms, as in the first phase. Following the termination of the second phase, the target stimulus was immediately presented for 500 ms. The change in orientation (from horizontal to vertical or from vertical to horizontal) corresponded to the deviant that violated a preceding sequential regularity, which was a repetition of an identical orientation from the beginning in the first phase. In this manipulation of the orientation, the colors for both eyes were not changed. As illustrated in Figure 1B, the target stimulus yielded three conditions dependent on the subject’s conscious/unconscious percept just before itself: the Standard (STD) condition, the Unconscious-deviant (Uncon-DEV) condition, and the Conscious-deviant (Con-DEV) condition. In the STD condition, the target stimulus was the same as the grating images used in the second phase, except for their durations (no change in orientation). In the Uncon-DEV condition, the orientation of the grating, which appeared “unconsciously,” was changed by 90°. In the Con-DEV condition, the orientation of the grating, which was perceived “consciously,” was changed by 90°. Which of the stimulus presented to both eyes changed in each trial had been determined, based on the perceptual report immediately prior to the target stimulus. The target stimulus was immediately followed by a cue image, which appeared for up to 3 s. In the cue image, the white fixation cross of the target stimulus was replaced with a green one for both eyes. When the green fixation appeared, participants were asked to stop pressing the left arrow key or the right arrow key immediately. Then, they were required to promptly report their currently perceived grating image again by pressing either the left arrow key or the right arrow key, as in the task during the second phase. Upon pressing the key, the cue image immediately disappeared. The inter-trial interval (ITI) was 2 s. During the ITI, the rings and the green fixation point were exclusively presented. Each of the three conditions contained 120 trials. The order of these stimulus conditions was randomized across trials. There were 8 sessions in the present study, each of which had 45 trials. Participants were given a rest between sessions, if needed.

### Analysis of Behavioral Data

Under the current stimulation paradigm, the number of trials in which the perceived color changed after the target stimulus in each condition was obtained. In this analysis, we first counted the trials in which participants kept pressing a response key for at least 500 ms just before the onset of the target stimulus. Due to latency of the behavioral response, the timing of the participants’ key press would lag from the perceived rivalry changes by approximately 450–500 ms (Alais et al., 2010). The current procedure was thus expected to extract behavioral data regarding whether participants kept their percepts for a certain period of time just before the onset of the target stimulus. We further narrowed the trials down to only those in which a participant stopped pressing a response key and pressed again following the onset of the cue image. In this procedure, the trials in which participants responded within 300 ms after the cue onset were excluded to ensure that included participants had checked the cue. Following these procedures, we obtained valid trials and counted the number of times when the perceived color changed from before to after the onset of the target stimulus; we then calculated the proportion of perceptual alternation for every condition. The calculated proportions of perceptual alternation were submitted to a repeated-measures one-way analysis of variance (ANOVA) with a factor of the conditions (the STD condition, the Uncon-DEV condition, and the Con-DEV condition). *Post hoc* tests were performed with Bonferroni correction. For the valid trials, we further evaluated whether the effects of the target stimulus on the proportion of the perceptual alternation would depend on a perceived grating prior to the presentation of the target stimulus. For each condition, in every participant, the proportion of perceptual alternation was calculated for each direction of perceptual change (i.e., perceptual changes from the blue grating to the red grating or vice versa). The proportion obtained were then submitted to a repeated-measures two-way ANOVA with factors of the directions of perceptual change and the condition. In the statistical analyses, the significance level was set at *p* < 0.05.

### Electroencephalography Recording

Electroencephalography (EEG) in each condition was recorded by the measurement instrument with 57 electrodes (EEG-1200, Nihon Kohden, Tokyo, Japan; EasyCap GmbH, Herrsching, Germany). The layout of electrodes was based on a modified version of the international 10-20 system. Impedance at each electrode was kept at less than 10 kΩ. EEG signals were digitized at 1 kHz and recorded with a 0.5–300 Hz band-pass filter online. For data acquisition, EEG signals were referenced to the right earlobe, and eye movement was monitored using horizontal and vertical bipolar electrooculograms (EOGs).

### Analysis of Electroencephalography Data

Electroencephalography signals were low-pass filtered offline at 30 Hz. EEG epochs from 100 ms before to 500 ms after the onset of the target stimulus in valid trials were collected (see section “Analysis of Behavioral Data” for details). We then calculated the mean of the EEG epochs across trials to obtain VEPs which were time-locked to the target stimulus. In this calculation of VEPs, EEG epochs containing a deflection of greater than ±100 μV in at least one electrode, or of greater than 60 μV at EOGs, were excluded from averaging. With this procedure, at least 75 artifact-free EEG signals (mean ± SD, STD condition: 103.4 ± 12.5 trials, Uncon-DEV condition: 109.6 ± 8.41 trials, Con-DEV condition: 109.3 ± 10.5 trials) were averaged in each condition for each participant. The mean amplitude for a period of −100 to 0 ms relative to the stimulus onset was used as the baseline, and the obtained VEP was re-referenced to the average of all electrodes to be consistent with our previous studies (Urakawa et al., 2017a, 2018). Based on previous studies (e.g., Guthrie and Buchwald, 1991; Doniger et al., 2001; Urakawa et al., 2017a), the difference in the VEP amplitude at Oz electrode between the Con-DEV condition and the STD condition, as well as that between the Uncon-DEV condition and the STD condition, was, respectively, evaluated using a series of two-tailed *t*-tests through successive time points. When the *t*-tests exceeded the 0.05 criterion for at least 20 subsequent time points, the amplitude difference between the conditions was considered to be significant. To record vMMN evoked by the deviant presented under the unconscious condition (Uncon-vMMN) as well as that evoked by the deviant presented under the conscious condition (Con-vMMN), VEP to the target stimulus for the STD condition was subtracted from that to the target stimulus for the Uncon-DEV condition or from that to the target stimulus for the Con-DEV condition. In line with our previous studies (Urakawa et al., 2017a, 2018), vMMNs prominently appeared at Oz across participants for both the Uncon-vMMN and the Con-vMMN. The present study thus focused on vMMN at Oz. By using differential VEPs (Uncon-DEV/Con-DEV – STD) at Oz, we visually identified their negative peaks at the latencies 100 ms later than the target stimulus’s onset. vMMN’s latencies/amplitudes were then obtained; the first negative peak was identified as vMMN1’s peak and the second negative peak was identified as vMMN2’s peak. The difference in vMMN’s peak latency/amplitude between the Uncon-vMMN and the Con-vMMN was evaluated using paired *t*-tests.

### Correlation Analysis

As in previous studies (Urakawa et al., 2017a, 2018), we further performed correlation analyses between the differential proportion of perceptual alternation (Uncon-DEV – STD or Con-DEV – STD) and peak latency/amplitude of vMMN across participants. The differential proportion was calculated by subtracting the proportions of perceptual alternation between the conditions, Uncon-DEV and STD or Con-DEV and STD. In the correlation analysis, absolute values of vMMN’s amplitude were evaluated. Such correlation analyses between behavioral index and neural data, with a focus on inter-individual differences, is expected to be a powerful approach in deducing a neural mechanism underlying behavioral data (e.g., Vogel and Awh, 2008). The Spearman’s rank order correlation coefficient was calculated. In the analyses, vMMN data that were not clearly identified upon visual inspection were excluded, and the significance level was set at *p* < 0.05.

## Results

### Behavioral Data

**FIGURE 2:**
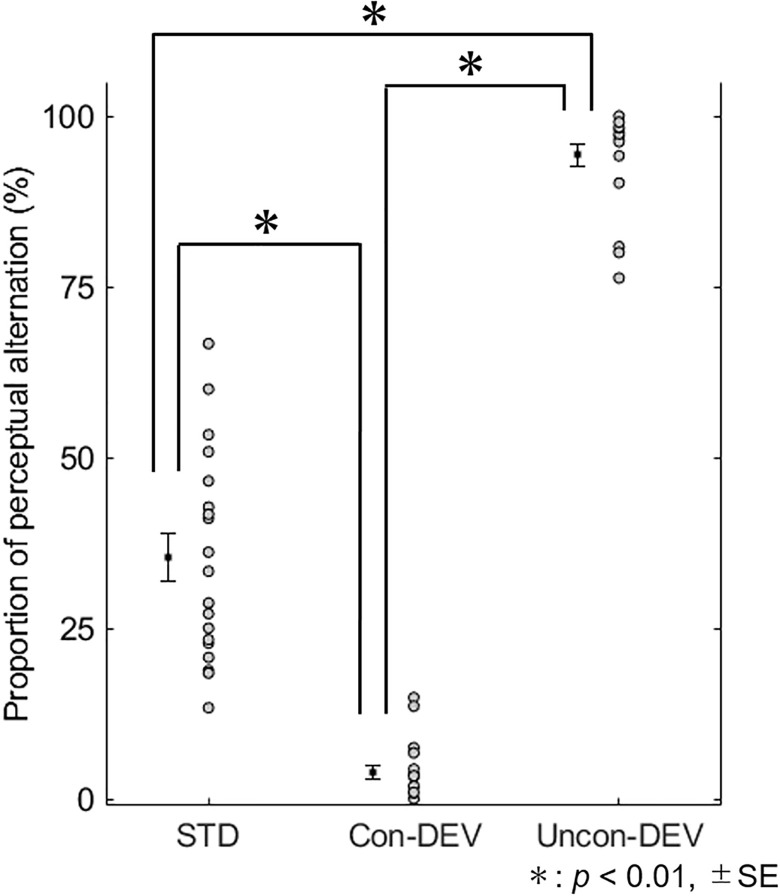
Proportion of perceptual alternation for each condition. The proportion of perceptual alternation for all participants are shown for each condition. The mean proportion is indicated by black-filled square with ±SE. The proportion was significantly higher in the Uncon-DEV condition than that in the STD condition. On the contrary, the proportion was significantly lower in the Con-DEV condition than that in the STD condition.

Figure 2 shows the proportion of perceptual alternation from before to after target stimulus. The mean proportions of perceptual alternation were 0.353 ± 0.035 (SE) for the STD condition, 0.943 ± 0.016 (SE) for the Uncon-DEV condition, and 0.039 ± 0.010 (SE) for the Con-DEV condition. For each condition, no value of the proportion exceeded the range of the mean ± 3 SD. A repeated-measures one-way ANOVA revealed that the proportion was significantly affected by the conditions [*F*(2,36) = 424.023, *p* < 0.01, $\eta_{\text{p}}^{2}$ = 0.959]. An analysis of multiple comparisons further revealed that the Proportion in the Uncon-DEV condition was significantly higher than that in the STD condition [*t*(18) = 15.39, *p* < 0.01, Cohen’s *d* = 4.95, a *post hoc* test with a Bonferroni correction] and that the proportion in the Con-DEV condition was significantly lower than that in the STD condition [*t*(18) = 10.08, *p* < 0.01, Cohen’s *d* = 2.81, a *post hoc* test with a Bonferroni correction]. There was also a significant difference in the proportion between the Uncon-DEV condition and the Con-DEV condition [*t*(18) = 38.87, *p* < 0.01, Cohen’s *d* = 15.43, a *post hoc* test with a Bonferroni correction]. These results indicate that the deviant administrated on “an unconsciously presented image” is likely to render the unconscious image consciously perceived (i.e., the facilitation of the perceptual alternation) (Walker and Powell, 1979). In contrast, the deviant administrated on “a consciously presented image” operated on a percept of the image such that the image was consciously perceived from before to after the presentation of the deviant (i.e., the suppression of perceptual alternation). In further analysis, we evaluated whether the proportion of perceptual change would be affected by a perceived grating prior to the presentation of the target stimulus. For the STD condition, the mean proportion of a perceptual change from the blue grating to the red grating across participants was 0.210 ± 0.027 (SE), while that from the red grating to the blue grating was 0.143 ± 0.021 (SE). For the Uncon-DEV condition, the mean proportion of the perceptual change from the blue grating to the red grating was 0.446 ± 0.029 (SE), while that from the red grating to the blue grating was 0.498 ± 0.031 (SE). For the Con-DEV condition, the mean proportion of the perceptual change from the blue grating to the red grating was 0.014 ± 0.004 (SE), while that from the red grating to the blue grating was 0.024 ± 0.007 (SE). A repeated-measures two-way ANOVA revealed that the proportion was not significantly affected by the direction of perceptual change [*F*(1,18) = 0.005, *p* = 0.946, $\eta_{\text{p}}^{2}$ < 0.01]. There was no significant interaction between the conditions and the direction of perceptual change [*F*(2,36) = 2.336, *p* = 0.111, $\eta_{\text{p}}^{2}$ = 0.115]. These findings indicate that the proportion of perceptual alternation was insusceptible to changes in consciously-perceived color from before to after the onset of the target stimulus.

### Electroencephalography Data

**FIGURE 3:**
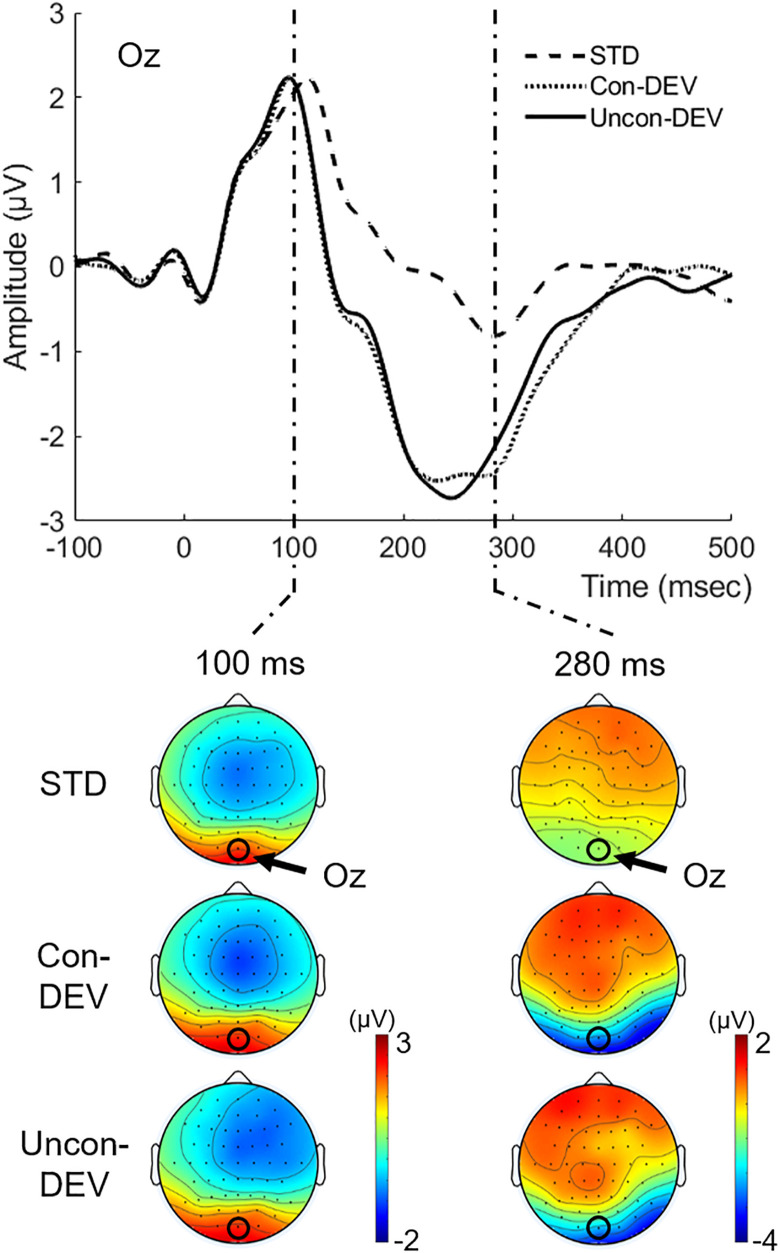
Grand-averaged VEPs to the target image for each condition. VEPs at Oz and their isocontour maps at latencies of 100 and 280 ms were illustrated for each condition. VEPs in both the Con-DEV condition and the Uncon-DEV condition were more negatively enhanced than VEP in the STD condition at a latency range of approximately 100–400 ms.

Figure 3 shows the grand-averaged VEP waveform at Oz and isocontour maps at latencies of 100 and 280 ms for each condition. The VEP amplitude appeared to be more negatively shifted for the Uncon-DEV condition or the Con-DEV condition than for the STD condition at Oz. Two-tailed *t*-tests through successive time points (see section “Materials and Methods” for details) revealed that the negative shift of VEP for the Uncon-DEV condition over the STD condition was significant at a latency of 115–334 ms. The enhancement of VEP for the Con-DEV over the STD condition was also significant at a latency of 114–362 ms.

**FIGURE 4:**
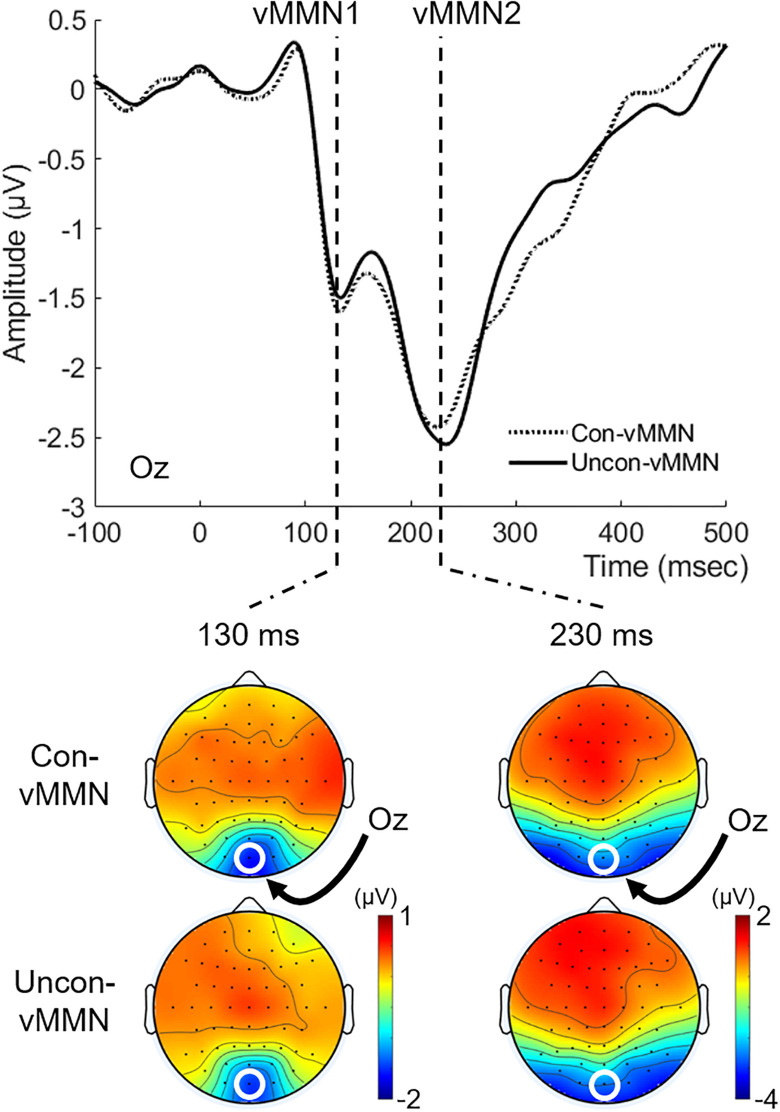
Grand-averaged vMMNs to the target image. vMMN at Oz and topographical maps at latencies of 130 and 230 ms were shown for each condition. Two successive vMMNs (vMMN1 and vMMN2, see “Electroencephalography Data” section) emerged. vMMNs evoked in both the Uncon-DEV condition and the Con-DEV condition prominently appeared at Oz.

Figure 4 shows the grand-averaged vMMNs (Con-vMMN and Uncon-vMMN) at Oz with their isocontour maps at latencies of 130 and 230 ms. In line with our previous studies (Urakawa et al., 2017a, 2018), the negative shift appeared at Oz particularly at a latency range of approximately 100–400 ms. As in previous studies (e.g., Maekawa et al., 2005), two successive peaks for posterior negativities (hereafter, we refer to the first negativity as vMMN1 and the second negativity as vMMN2) were observed at a latency of approximately 100–250 ms for both the Uncon-vMMN and the Con-vMMN. These findings support the fact that our current stimulation paradigm was effective in evoking vMMN.

For each vMMN1 and vMMN2, we evaluated the difference in the vMMN peak latency/amplitude between the Uncon-vMMN and the Con-vMMN. The vMMN2 data for three participants were excluded from analysis due to lack of prominent vMMN2 emergence. For vMMN1, the mean of peak latency was 145 ± 7.3 (SE) ms in the Uncon-vMMN and 147 ± 7.5 (SE) ms in the Con-vMMN. The mean of peak amplitude was −2.636 ± 0.356 (SE) μV in the Uncon-vMMN and −2.621 ± 0.315 (SE) μV in the Con-vMMN. Paired *t*-tests indicated that there was no significant difference in both peak latency and peak amplitude between the Uncon-vMMN and the Con-vMMN [for peak latency, *t*(18) = 0.832, *p* = 0.416, Cohen’s *d* = 0.05; for peak amplitude, *t*(18) = 0.089, *p* = 0.930, Cohen’s *d* = 0.01]. As for vMMN2, the mean of peak latency was 237 ± 5.1 (SE) ms in the Uncon-vMMN and 245 ± 6.6 ms (SE) in the Con-vMMN. Paired *t*-tests indicated that the Uncon-vMMN was significantly elongated in latency over the Con-vMMN [*t*(15) = 2.228, *p* = 0.042, Cohen’s *d* = 0.35]. The mean of peak amplitude was −2.777 ± 0.412 (SE) μV in the Uncon-vMMN and −2.664 ± 0.340 (SE) μV in the Con-vMMN. There was no significant difference in amplitude between the Uncon-vMMN and the Con-vMMN [*t*(15) = 0.523, *p* = 0.609, Cohen’s *d* = 0.07].

### Correlation Between Behavioral Data and Visual Mismatch Negativity

**FIGURE 5:**
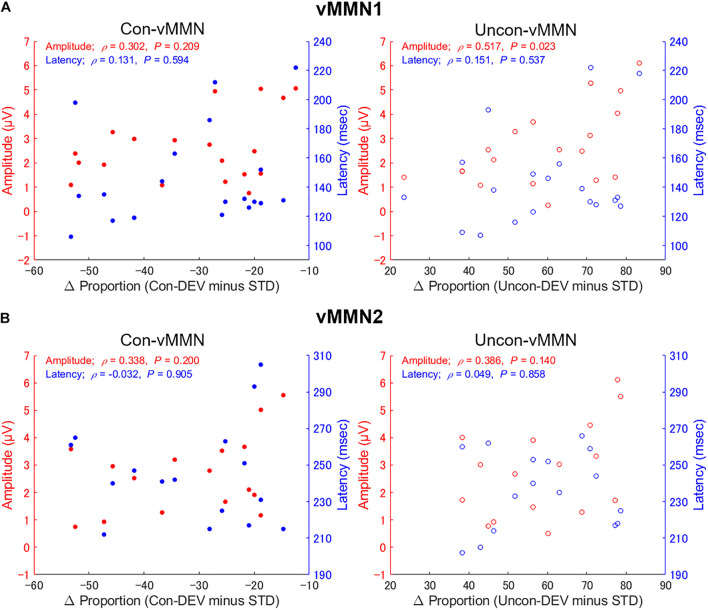
Relationship between proportion of perceptual alternation and vMMNs across participants. The correlations between the differential proportion of perceptual alternation (Uncon-DEV condition – STD condition; Con-DEV condition – STD condition) and vMMN (Uncon-vMMN; Con-vMMN) are shown for vMMN1 **(A)** and vMMN2 **(B)**. There was a significant correlation between differential proportion (Uncon-DEV condition – STD condition) and an enhancement of the Uncon-vMMN, indicating that the Uncon-vMMN is relevant to making an unconsciously presented image consciously perceived.

With a focus on inter-individual variability in behavioral and neural data, we evaluated whether the Uncon-vMMN and the Con-vMMN would be relevant to the facilitation and suppression of the perceptual alternation, respectively. Figure 5A shows the results of correlation analyses for vMMN1. In the Con-vMMN, there was no significant relationship between the differential proportion of perceptual alternation and latency [ρ(19) = 0.131, *p* = 0.594] or amplitude [ρ(19) = 0.302, *p* = 0.209] across participants. As for the Uncon-vMMN, there was not a significant correlation between the differential proportion of perceptual alternation and latency [ρ(19) = 0.151, *p* = 0.537], there was a significant positive correlation between differential proportion and amplitude [ρ(19) = 0.517, *p* = 0.023], with an enhancement of the Uncon-vMMN significantly correlated with facilitation of the perceptual alternation across participants. Figure 5B shows the results of correlation analyses for vMMN2. In the Con-vMMN, there was no significant relationship between differential proportion and latency [ρ(16) = −0.032, *p* = 0.905] or amplitude [ρ(16) = 0.338, *p* = 0.200]. As for the Uncon-vMMN, there was not significant correlation between differential proportion and latency [ρ(16) = 0.049, *p* = 0.858] or amplitude [ρ(16) = 0.386, *p* = 0.140]. Considering these results, the peak amplitude of vMMN1 for the Uncon-DEV condition exclusively reflects perceptual alternation in a manner that the enhancement of the vMMN (the Uncon-vMMN) is relevant to rendering an unconsciously presented image perceived consciously. The correlation between Uncon-vMMN and perceptual alternation in the present study was consistent with those in the previous studies (Urakawa et al., 2017a, 2018). The number of participants for evaluating the individual differences in the present study was also comparable to the previous studies (10–20 participants).

The VMMN was evoked by conscious and unconscious deviant stimuli. Our behavioral results showed that the conscious deviant stimulus suppressed perceptual alternation, while the unconscious deviant stimulus facilitated perceptual alternation. These EEG and behavioral results suggest that vMMN may be involved in making the deviant stimulus more perceptible, independent of whether the deviant was presented consciously or unconsciously. On the other hand, there was a significant correlation between the behavioral index and vMMN in the unconscious condition, although there was no significant difference in the conscious condition. These results indicate that the neural processing underlying vMMN is closely involved in the visual perceptual processing of the unconscious stimulus rather than the conscious stimulus. Thus, vMMN is related to APVA, which is an unconscious neural process.

## Discussion

The present study confirmed the hypothesis that vMMN reflecting the automatic visual change detection is relevant to APVA. The deviant stimulus of orientation change enabled us to discover the relationship between the perceptual alternation and vMMN. Consequently, we found a significant correlation between the enhancement of Uncon-vMMN’s amplitude and the facilitation of perceptual alternation when the unconscious deviant was presented. On the other hand, no significant correlation was observed when the conscious deviant was presented. These results indicated that the unconscious visual processing underlying vMMN is involved in APVA.

Our current finding showed that the unconscious deviant, which was an external perturbation, made the unconscious stimulus more likely to be consciously perceived. This might originate from exogenous attention induced by the unconscious deviant. This reasoning is consistent with previous studies, which showed that exogenous attention to a certain location of the visual field facilitates visual processing to the invisible target image presented at the location; thus, the exogenous attention plays an important role in shaping conscious perception (Chica et al., 2010, 2011). Taken together, these results support our hypothesis that vMMN evoked by the unconscious deviant and its associated attentional mechanism make it easier for the unconscious stimulus to be consciously perceived.

In our experimental paradigm, the orientation of the target stimulus was the same as that of the preceding images for the STD condition. Meanwhile, for the DEV conditions (Con-DEV condition and Uncon-DEV condition), the orientation of the target stimulus differed from that of the preceding images. In such a stimulation method, vMMN’s emergence was expected to be partly ascribed to neural refractoriness for the orientation in the STD condition. In this regard, vMMN recorded in the present study was not genuine vMMN which is obtained by controlling neural adaptation/refractoriness to the standard. In the predictive coding framework, neural adaptation/refractoriness reflects a decrease in prediction error and neural activity’s enhancement for the deviant mirrors an increase in prediction error (e.g., Friston, 2005; Stefanics et al., 2014). From this perspective, our current finding (the significant correlation between vMMN’s amplitude and the facilitation of perceptual alternation) may indicate that an increase of the prediction error invoked under the unconscious condition is relevant to the facilitation of APVA.

The previous study (Jack et al., 2017) reported that vMMN is evoked by the unconsciously presented deviant of decremental luminance, but it was impossible to evaluate whether vMMN affects subsequent conscious perception or not. This is because such a deviant, defined as a luminance decrement, would suppress perceptual alternation (Levelt, 1965); that is, the deviant with luminance decrement would suppress APVA. By adopting a stimulus which did not suppress perceptual alternation as the deviant, the present study showed that vMMN is not only evoked by the unconscious stimulus, but is also involved in APVA, which involves an unconscious neural processing after emergence of vMMN. In our previous studies, we reported that vMMN is relevant to exogenously driven perceptual alternation on the Necker cube (Urakawa et al., 2017a, 2018). The present study additionally suggests that the automatic visual change detection underlying vMMN is also related to APVA.

In perceptual alternations of bistable perception, such as the Necker cube and binocular rivalry, VEPs time-locked to the image that induce perceptual alternation are known to be more negatively shifted over posterior electrodes at a latency of about 150–250 ms than when perceptual alternation does not occur (Kornmeier and Bach, 2004; Britz and Pitts, 2011). This negative enhancement in VEP amplitude is called reversal negativity (RN). Since the perceptual alternation in the Uncon-DEV condition was more enhanced than the STD condition in our study, there is a possibility that the Uncon-vMMN contained an RN component. On the other hand, a previous study reported a negative correlation between RN magnitude and the number of perceptual alternations across participants (Russo and De Pascalis, 2016), while our results show a positive correlation between Uncon-vMMN’s amplitude and the proportion of perceptual alternation. Thus, if we assume that the enhancement of Uncon-vMMN is mainly due to the effect of RN, the positive correlation reported in the present study is contradictory to the previous report. Therefore, the relevance of vMMN to APVA is suggested, despite the possibility of RN contamination.

Concerning vMMN2, the peak latency of Uncon-vMMN2 was significantly longer than that of Con-vMMN2 (see section “Electroencephalography Data” in Results). In vMMN latency, we should take VAN into account. This is because VAN is observed at a peak latency of approximately 200 ms after the stimulus onset when the visual stimulus is consciously perceived (Koivisto and Revonsuo, 2003; Koivisto and Grassini, 2016). In the present study’s results, the proportion of perceptual alternation in the Uncon-DEV condition was very high, at about 95%, and the unconscious visual stimulus was almost consciously perceived. Therefore, it is highly likely that VAN, which is related to the conscious perception of the visual stimulus, emerged in the Uncon-vMMN2 and affected peak latency.

In the global neuronal workspace (GNW) framework, conscious perception correlates with the global neuronal workspace, which links features represented in different brain areas and binds them into coherent representations, and later brain activities (P3 or late-positive component) reflect conscious perception (Roelfsema et al., 2000; Sergent et al., 2005; Dehaene and Changeux, 2011; Mashour et al., 2020). On the other hand, our study focused on pre-conscious visual processing for less than 200 ms evoked by a mismatch stimulus. In GNW, attention plays an important role in selecting a piece of information by amplifying its activity and reducing that of other competing stimuli, with this attentional selection leading to conscious perception. Similarly, the present study suggested that the attention induced by the unconscious stimulus contributes to the selection of conscious perception. However, it is impossible to discuss further the detail of attention because we did not control the top-down attention accompanied with vMMN in the experiment. This must be investigated in the future studies.

Although the present study argued that the visual processing underlying vMMN is relevant to APVA, there remains a possibility that vMMN at least partly reflected neural activity concomitant with APVA. This is because the correlation in the present study did not necessarily imply causality. For example, the vMMN recorded in the present study might have been confounded by neural activity irrelevant to APVA. However, the main finding that vMMN is involved in APVA is unaffected by possible contamination because their effects are limited. To the best of our knowledge, vMMN preceding VAN was shown to be relevant to APVA for the first time.

In conclusion, the results showed a correlation between the enhancement of vMMN amplitude and the facilitation of perceptual alternation in binocular rivalry when an unconscious deviant was presented. This implies that vMMN, which reflects an automatic visual change detection, is relevant to APVA. In early visual processing, the attentional mechanism associated with vMMN is suggested to play an important role in APVA. The discovered relevance of vMMN on APVA is a significant first step in elucidating early unconscious processing before established conscious perception.

## Data Availability Statement

The original contributions presented in the study are included in the article/supplementary material, further inquiries can be directed to the corresponding author.

## Ethics Statement

The studies involving human participants were reviewed and approved by the Ethics Committee of the Tokyo University of Science. The patients/participants provided their written informed consent to participate in this study. Written informed consent was obtained from the individual(s) for the publication of any potentially identifiable images or data included in this article.

## Author Contributions

YK, TU, and OA designed the study. YK collected and analyzed data. YK drafted the manuscript. TU and OA edited and revised the manuscript. All authors contributed to the article and approved the submitted version.

## Conflict of Interest

The authors declare that the research was conducted in the absence of any commercial or financial relationships that could be construed as a potential conflict of interest.

## Publisher’s Note

All claims expressed in this article are solely those of the authors and do not necessarily represent those of their affiliated organizations, or those of the publisher, the editors and the reviewers. Any product that may be evaluated in this article, or claim that may be made by its manufacturer, is not guaranteed or endorsed by the publisher.
